# Characterization of a novel sugar transporter involved in sugarcane bagasse degradation in *Trichoderma reesei*

**DOI:** 10.1186/s13068-018-1084-1

**Published:** 2018-04-02

**Authors:** Karoline M. V. Nogueira, Renato Graciano de Paula, Amanda Cristina Campos Antoniêto, Thaila F. dos Reis, Cláudia Batista Carraro, Alinne Costa Silva, Fausto Almeida, Carem Gledes Vargas Rechia, Gustavo H. Goldman, Roberto N. Silva

**Affiliations:** 1 Department of Biochemistry and Immunology, Ribeirão Preto Medical School, University of São Paulo, Ribeirão Preto, SP Brazil; 2 Department of Pharmaceutical Sciences, Faculty of Pharmaceutical Sciences of Ribeirão Preto, University of São Paulo, Ribeirão Preto, SP Brazil

**Keywords:** *Trichoderma reesei*, Sugarcane bagasse, Cellulase, Sugar transporter, Docking, *Saccharomyces cerevisiae*, Biofuel

## Abstract

**Background:**

*Trichoderma reesei* is a saprophytic fungus implicated in the degradation of polysaccharides present in the cell wall of plants. *T. reesei* has been recognized as the most important industrial fungus that secretes and produces cellulase enzymes that are employed in the production of second generation bioethanol. A few of the molecular mechanisms involved in the process of biomass deconstruction by *T. reesei*; in particular, the effect of sugar transporters and induction of xylanases and cellulases expression are yet to be known.

**Results:**

In our study, we characterized a novel sugar transporter, which was previously identified by our group through in silico analysis of RNA-seq data. The novel *T. reesei* 69957-sugar transport system (*Tr69957*) is capable of transporting xylose, mannose, and cellobiose using a *T. reesei* 69957-sugar transport system in *Saccharomyces cerevisiae*. The deletion of *Tr69957* in *T. reesei* affected the fungal growth and biomass accumulation, and the sugar uptake in the presence of mannose, cellobiose, and xylose. Molecular docking studies revealed that *Tr69957* shows reduced protein–ligand binding energy for interactions towards disaccharides in comparison with monosaccharides. Furthermore, the deletion of *Tr69957* affected the gene expression of cellobiohydrolases (*cel7a* and *cel6a*), β-glucosidases (*cel3a* and *cel1a*), and xylanases (*xyn1* and *xyn2*) in the cultures of parental and mutant strains in the presence of cellobiose and sugarcane bagasse (SCB).

**Conclusion:**

The transporter *Tr69957* of *T. reesei* can transport cellobiose, xylose, and mannose, and can affect the expression of a few genes encoding enzymes, such as cellulases and xylanases, in the presence of SCB. We showed for the first time that a filamentous fungus (*T. reesei*) contains a potential mannose transporter that may be involved in the degradation of cellulose.

**Electronic supplementary material:**

The online version of this article (10.1186/s13068-018-1084-1) contains supplementary material, which is available to authorized users.

## Introduction

The fungus *Trichoderma reesei* is one of the most studied microorganisms and is known for its ability to degrade lignocellulose [1]. *T. reesei* has an efficient degradation system for major polysaccharides present in the plant cell wall, and thus, it is commercially exploited for its effective production and secretion of a wide range of cellulases, hemicellulases, and other Carbohydrate-Active Enzymes (CAZymes) [1, 2]. The expression of CAZymes-encoding genes occurs in a carbon-source-dependent manner and may be regulated by several transcription factors and small molecules that mediate the control of gene expression [3]. Moreover, a wide diversity of mono- and disaccharides is naturally found during biomass hydrolysis, being able to act as regulatory molecules [1, 4]. Accordingly, a complex carbon source such as sugarcane bagasse (SCB), which is the major residue form the Brazilian agroindustry and constituted by cellulose (40–50%), hemicellulose (25–35%), and lignin (15–20%), can be efficiently degraded by fungal enzymes to release key regulatory molecules that might in turn activate the expression of genes to improve the sensing of different carbon sources and secretion of cellulolytic enzymes [5, 6].

The access to sugars released from the complex polysaccharides is dependent on the ability of this fungus to secrete high amounts of a complex mix of hydrolytic enzymes, and also the presence of a large array of sugar transporters that are capable of effectively transporting the constituent sugars into the cell [7]. Therefore, the transporters are essential in the utilization of lignocellulose by the fungus, promoting the transport of molecules that signal environmental changes to the cell and, consequently, controlling the CAZymes-encoding gene expression [1]. One of the most relevant sugar transporters families in filamentous fungi is the Major Facilitator Superfamily (MFS) [8]. In the *T. reesei* genome, approximately 164 predicted MFS transporters have been annotated [9]. However, the involvement of these transporters in sugar uptake is still unclear, and only a few sugar transporters have been functionally characterized in filamentous fungi. Interestingly, the available data show that MFS transporters can recognize and transport more than one type of sugar such as xylose and cellobiose into the cell [10]. For instance, *T. reesei* STP1 is involved in glucose and cellobiose uptake [8]. Moreover, the *A. nidulans* transporter XtrD was shown to be able to transport several other monosaccharides, in addition to xylose and glucose [11]. After the uptake, these sugars have been shown to play an important role in metabolic signaling in the presence of cellulose [12]. Despite the progress made in studies related to cellulases production, the influence of these transporters on cellulose degradation from various carbon sources remains unknown.

Our group has demonstrated new components involved in cellulose degradation including some transporters. A few of them that were identified in the transcriptome analysis were specifically induced by cellulose, sophorose, and glucose, whereas the MFS family was overrepresented in our analysis in the presence of different carbon sources [3]. Therefore, the aim of this study was to characterize one of the *T. reesei* transporters that was initially identified by RNA-seq and in silico analysis, and to understand its involvement in SCB degradation. We have characterized a *T. reesei* transporter with potential role in the transport of monosaccharides and disaccharides. The characterization of MFS *Tr69957* showed that this transporter can better enhance the efficiency of cellobiose, xylose, and mannose transport as compared to that by *T. reesei* mutant strain. In addition, with respect to the cellulases production, it was observed that the deletion of MFS *Tr69957* leads to decreased expression of cellulase and xylanase genes as compared to that in the parental strain when both the strains were grown on SCB. Furthermore, for the first time, our results showed that *T. reesei* possesses a potential mannose transporter, which may be involved in the degradation of cellulose.

## Materials and methods

### Fungal strains, media, and culture methods

The fungal strains used in this study are listed in Additional file 1. The *S. cerevisiae* strain EBY.VW4000 [13] is a hexose-null mutant and it was used for the initial functional analysis of the *Tr69957* transporter. The EBY.VW4000 and its mutants (EBY.VW4000 +pRH195m +pRH274 and *Tr69957*::GFP EBY.VW4000) were grown at 30 °C in broth or on solid yeast nitrogen base (YNB) medium (which contained 20 g/L agar, 7 g/L YNB without amino acids and supplemented with 0.05 g/L histidine, 0.1 g/L leucine, 0.1 g/L tryptophan, and 0.1 g/L uracil). Selection of transformants was done on the YNB medium lacking tryptophan and uracil. All the reagents were obtained from Sigma Aldrich (St. Louis, MO, USA). The yeast *S. cerevisiae* SC9721 strain (*MATα his3*-*Δ200 URA3*-*52 leu2Δ1 lys2Δ202 trp1Δ63*) (Fungal Genetic Stock Center—www.fgsc.net) was used to generate the deletion cassette by homologous recombination. This strain was grown at 30 °C in broth or on solid YNB medium (which contains 20 g/L agar, 7 g/L YNB without amino acids and supplemented with 0.05 g/L histidine, 0.1 g/L lysine, 0.1 g/L leucine, 0.1 g/L tryptophan, 0.1 g/L uridine, and 0.1 g/L uracil), along with 2% (w/v) glucose. The strain QM6a∆*tmus*53∆*Pyr4* [14] was used as a parental strain to construct the ∆*69957 T. reesei* mutant strain. This strain was obtained from the Institute of Chemical Engineering & Technical Biosciences of Vienna University of Technology, TU Vienna, Austria. The strain was maintained at 4 °C on MEX medium [3% (w/v) malt extract and 2% (w/v) agar–agar], which was supplemented with 5 mM uridine in the case of the *pyr4* deletion strain. To perform gene expression assays, 10^6^ cells/mL of each specific strain was inoculated into 25 mL of Mandels–Andreotti medium [15] (MAM) or Minimal Medium (MM) which contains 1% (w/v) of the selected sugar as the sole carbon source.

### Conditions for maintenance of cultures

*Trichoderma reesei* strains QM6a∆*tmus53*∆*Pyr4* [14] and ∆*69957* were grown in MEX medium at 28 °C for a period of 7 days. In all the experiments with SCB, the parental and Δ*69957* mutant strains were initially grown in 1% (w/v) glycerol for 24 and 48 h, respectively, and then transferred to a medium containing SCB. For this purpose, 10^6^ cells/mL of each specific strain was inoculated into 25 mL of MAM [15] containing 1% glycerol, and after 24 h (parental strain), the mycelium was transferred into 25 mL of MAM containing 1% of exploded sugarcane bagasse (ESB). Concerning the Δ*69957* mutant strain, the mycelium was initially grown in 1% (w/v) glycerol for 48 h to achieve an initial mycelial biomass similar to the parental strain, and then, the mycelium was transferred into 25 mL of MAM containing 1% ESB. The ESB was prepared as described previously in the corresponding Ref. [16]. Briefly, SCB *in natura* was treated with 14 kg/cm^2^ water steam, thoroughly washed with distilled water until the reducing sugars were not detected by dinitrosalicylic acid (DNS) [17], and dried at 40 °C for several days. SCB was kindly donated by Nardini Agroindustrial Ltd, Vista Alegre do Alto, São Paulo, Brazil.

The fungal cultures were incubated at 28 °C for 6, 24, and 48 h in an orbital shaker (200 rpm) for all the SCB experiments. All experiments were performed in three biological replicates. The resultant mycelia were collected by filtration, frozen in liquid nitrogen, and stored at − 80 °C for RNA extraction.

### Construction of the ∆*69957* mutant strain

The deletion of Tr69957 gene in *T. reesei* was performed as described previously by Schuster et al. [18]. To construct the deletion cassette, the orotidine-5′-phosphate decarboxylase gene of *Aspergillus niger* (*pyrG*) was used as a selection marker. The flanking sequences of *Tr69957* were obtained from the *T. reesei* genome database (http://genome.jgi.doe.gov/Trire2/Trire2.home.html). The marker gene and its respective 5′ and 3′ flanking sequences were amplified using the primers as described in Additional file 2. The primers were designed and analyzed with the help of OligoAnalyzer tool (https://eu.idtdna.com/calc/analyzer). To enable yeast-mediated recombination of the deletion cassette, we used the external 5′-UTR forward and 3′-UTR reverse primers containing cohesive ends with the vector pRS426 [19, 20] and the internal 5′-UTR reverse and 3′-UTR forward primers containing cohesive ends with the 5′ and 3′ sequence of the *pyrG* gene (Additional file 2). The 50-μL reaction mixture contained 1 U Platinum Taq DNA Polymerase High Fidelity (Thermo Scientific), 1 × High Fidelity PCR Buffer, 0.2 mM dNTPs, 0.3 µM forward and reverse primers, 1 µL *T. reesei* QM9414 and *A. niger* genomic DNA (150 ng/µL) as a template, and nuclease-free water. The PCR fragments were purified using a QIAquick PCR Purification Kit (Qiagen).

To perform yeast-mediated recombination, we used the yeast shuttle vector pRS426 (*amp*^R^ *lacZ* URA3) [19, 20], which was digested with *Eco*RI and *Xho*I (Thermo Scientific), and purified with the QIAquick PCR Purification Kit (Qiagen). Yeast transformation was performed as described previously in the corresponding Ref. [21–23]. We prepared an overnight culture (200 rpm, 30 °C) of the yeast *S. cerevisiae* SC9721 strain (*MATα his3*-*Δ200 URA3*-*52 leu2Δ1 lys2Δ202 trp1Δ63*) obtained from Fungal Genetic Stock Center. Then, 1 mL of this overnight culture was added to 50 mL of fresh YPD medium (1% yeast extract, 2% peptone, 1% glucose: all of them were obtained from Sigma Aldrich) and incubated at 30 °C until the attainment of OD_600_ = 1. Thereafter, the cells were centrifuged and re-suspended in 100 mM lithium acetate for transformation. To this purpose, equimolar quantities of 5′ and 3′ flanking regions of *pyrG* gene and the digested pRS426 product were mixed and used for yeast transformation by the lithium acetate method [24]. *S. cerevisiae* SC9721 transformants were selected for their ability to grow on YPD medium supplemented with lysine, histidine, leucine, and tryptophan, without uracil. *S. cerevisiae* genomic DNA was extracted using a previously described protocol [25, 26]. The deletion cassette of Tr*69957* was PCR amplified using TaKaRa Ex Taq DNA Polymerase (Clontech) using *Tr69957*_pRS426_5 forward and *Tr69957*_pRS426_3 reverse primer (Additional file 2).

### Southern blotting

The selected transformants were analyzed by Southern hybridization using a previously described method [27], to demonstrate that the transformation cassettes had homologously integrated at the targeted *T. reesei QM6aΔtmus53Δpyr4* loci (Additional file 3). To perform this analysis, 25 µg of total genomic DNA from the parental and mutant strains were digested with *Eco*RV (Fermentas) overnight, and then, this digested DNA was transferred onto GE Healthcare Amersham Hybond-N + membranes (GE). The probe was generated from the PCR-amplified fragment using *Tr69957*_pRS426_5fw and *Tr69957*_*pyrG*_5rv (Additional file 2) and labeled using a digoxigenin (DIG) DNA labeling kit (Roche, Mannheim, Germany) by following the manufacturer’s instructions. Labeling, hybridization, and immunological detection were performed using a non-radioactive labeling and immunological detection kit with CDP-Star as the chemiluminescent substrate (Roche, Mannheim, Germany), by following the methods that were previously described [28].

### Measurement of dry weight

A total of 10^6^ cells/mL of *T. reesei* parental or Δ*69957* mutant strains were inoculated into 25 mL of MAM supplemented with 1% (w/v) glycerol. After 24 h, the parental strain mycelium was transferred into 25 mL of MAM containing 1% (w/v) xylose, cellobiose, or mannose. The mutant strain was grown for 48 h in MAM containing 1% (w/v) glycerol to achieve an initial mycelial biomass similar to the parental strain. After 6, 24, and 48 h, mycelia were harvested by filtration, dried by incubation at 70 °C for 3 h and subsequently weighed. The experiments were conducted in triplicates for each sample.

### Determination of the extracellular sugar concentrations

The uptake of sugars was measured by HPLC analyses. A total of 10^6^ cells/mL *T. reesei* parental and Δ*69957* mutant strains were inoculated into 25 mL of MAM supplemented with 1% (w/v) glycerol. After 24 h, the parental strain mycelium was transferred into 25 mL of MAM containing 1% (w/v) or 30 mM of xylose, cellobiose, or mannose in the same culture, at 30 °C 200 rpm for 6, 24, and 48 h. The mutant strain was grown for 48 h in MAM containing 1% (w/v) glycerol to achieve an initial mycelial mass similar to that of the parental strain. *S. cerevisiae Tr69957*::GFP EBY.VW400 strain and EBY.VW4000 + pRH195m + pRH274 + pGH1 (control) were inoculated in 100 mL of YNB medium supplemented with 2% (w/v) maltose until they reached the exponential growth phase. Yeast cells were pelleted by centrifugation at 4000 rpm for 5 min, washed three times with 50-mL water, and re-suspended in water. Next, this cell suspension was inoculated (initial OD_600_ = 0.5) in 50-mL YNB-Trp-Ura-Leu medium without carbon source and then incubated at 30 °C for 3 h. Finally, this medium was supplemented with 1% (w/v) xylose, cellobiose, or mannose at 30 °C, 150 rpm for 144 h. At each timepoint, 2 mL of the culture were collected, centrifuged, and the supernatants were stored at − 80 °C. The amount of sugar in the supernatant at each timepoint was determined by ion chromatography (HPLC-Thermo Fisher-U3000), coupled with refractive index detector) Suplecogel C611 column (eluted with 5 mM H_2_SO_4_, at flow rate 0.5 mL/min and column temperature 60 °C) and Aminex HPX-87P column (eluted with H_2_O, at flow rate 0.5 mL/min and column temperature 85 °C).

### Analysis of the effect of *Tr69957* deletion on *T. reesei* growth in various carbon sources

To analyze the effect of the *Tr69957* deletion on the growth of *T. reesei* in various carbon sources, phenotypic assays for the parental and mutant strains were performed by inoculating of 10^6^ cells/mL on minimal media (MM) and potato dextrose agar (PDA) plates containing 1% of one of the following carbon sources: mannose, xylose, glucose, galactose, arabinose, raffinose, cellobiose, sophorose, lactose, xylitol, glycerol, fructose, or maltose [29]. The experiment was performed in triplicates for each carbon source. The diameters of colonies were measured after 6 days.

### Quantitative real-time PCR analysis

*Trichoderma reesei* mycelia were macerated and total RNA was extracted from each sample using TRIzol^®^ RNA kit (Invitrogen Life Technologies, Carlsbad, CA, USA), according to the manufacturer’s instructions. RNA concentration was determined using a spectrophotometer at an optical density ratio of 260/280 nm, and RNA integrity was verified by electrophoresis in 1% agarose gels. To perform the expression analysis, initially, the total RNA (1 µg) from each sample was digested with DNase I (Fermentas) to remove genomic DNA. Then, cDNA synthesis was carried out using the RevertAid H Minus First Strand cDNA Synthesis kit (Waltham, Massachusetts, USA) according to the manufacturer’s instructions. Synthesized cDNA was diluted at 1:50 and used as a template for real-time PCR. Reactions were performed in the Bio-Rad CFX96™ using SsoFast™ EvaGreen^®^ Supermix (Bio-Rad) for detection according to the manufacturer’s instructions. Each reaction mixture (10 µL) contained 5 µL SsoFast™ EvaGreen^®^ Supermix (Bio-Rad), forward and reverse primers (500 nm each; Additional file 4), cDNA template, and nuclease-free water. PCR cycling conditions: 10 min at 95 °C (1 cycle), 10 s at 95 °C followed by 30 s at 60 °C (40 cycles), and a melting curve of 60–95 °C with an increment of 0.5 °C/10 s to check for primer dimers and nonspecific amplification. The transcript of the *sar* gene (encodes Sar1 GTPase from *T. reesei*) was used as an internal reference to normalize the amount of total RNA present in each reaction [30]. To perform gene expression analysis in strains grown on SCB, cellobiose, mannose, and xylose, the expression levels of genes were calculated from the threshold cycle according to the 2^−ΔCT^ method [31] relative to transcription levels of *sar*. The experiment was repeated three times for each sample. The results were analyzed using MeV v.4.6.1. Software and heat maps were constructed to assess the variety in the expression of genes among the strains under each indicated condition.

### Construction of *S. cerevisiae* strain expressing *T. reesei Tr69957*

The construction of *S. cerevisiae* strain that expresses *T. reesei Tr69957* was performed as described by dos Reis et al. [44] using the EBY.VW4000 yeast strain (Additional file 1). Briefly, the *T. reesei Tr69957* ORF was PCR-amplified using cDNA obtained from the wild-type (WT) strain QM6A (Additional file 1) using specific primers *69957*-F and *69957*-R. To perform in vivo recombination, the plasmid pRH195 was linearized by double digestion with *Spe*I and *Sal*I, originating the pRH195m (pRH195 without *XKS1* gene). The *GFP* gene was PCR amplified from pCMC17apx plasmid using primers pRH195 GFP_CS_F-yeast and spacerGFP 5´R_pRH195-yeast. Furthermore, the linearized plasmid was co-transformed with the PCR-amplified sugar transporter and GFP fragments into the *S. cerevisiae* EBY.VW4000 strain, which already contained the pRH27412 and pGH1x plasmids. The pRH274 plasmid contains the xylulose kinase (*XK*), xylose reductase (*XR*), and xylose dehydrogenase (*XDH*) genes which encode enzymes of the xylose metabolic pathway while the pGH1 plasmid contains the β-glucosidase-encoding gene (*gh1*-*1)* from *Neurospora crassa* [32]. The yeast transformation was performed using the lithium acetate method [33]. Transformants were selected for tryptophan, leucine, and uridine prototrophy, and the yeast candidates were confirmed by PCR using the primers *69957*-F and *69957*-R. A list of all the primers used in this study can be accessed in Additional file 5.

### Growth of *S. cerevisiae* strain on solid medium

*Saccharomyces cerevisiae* strains were inoculated in 50 mL YNB medium supplemented with 2% (w/v) maltose for 48 h at 30 °C, 200 rpm. Yeast cells were centrifuged at 4000 rpm for 5 min, washed three times with water, and re-suspended in water to a final concentration of 1.0 at OD_600nm_. A serial dilution of 1:10 of the yeast cells was done, and 5 μL of the cell suspensions were spotted on plates containing 1% (w/v) of specific carbon source. Plates were incubated at 30 °C for 120 h.

### Microscopy

*Saccharomyces cerevisiae* EBY.VW400 strain containing the *Tr69957* gene tagged with GFP was grown for 48 h in 0.5 mL of liquid YNB-Trp-Ura-Leu medium supplemented with 2% (w/v) maltose for 24 h at 30 °C in a well of 24-wells plate. Cells were washed with PBS and observed under a Carl Zeiss (Jena, Germany) microscope using the 100 × magnification oil immersion objective lens (EC Plan-Neofluar, NA 1.3) equipped with a 100-W HBO mercury lamp epifluorescence module. Phase contrast bright-field and fluorescent images were acquired with an AxioCam camera (Carl Zeiss), and images were processed using the AxioVision software version 3.1 and saved as TIFF files.

### 3-D structure prediction and molecular docking

The 3D model for *Tr69957* was built using the I-TASSER online platform from the Yang Zhang’s Research Group (https://zhanglab.ccmb.med.umich.edu/I-TASSER/). To this purpose, an FASTA file containing the amino-acid sequence for the referred protein was obtained from the UniProt database (gene: TRIREDRAFT_69957) and submitted to the I-TASSER online server. The structure modeling approach used by this software is based on the sequence alignment to a protein template, which is identified by LOMETS, a method that selects the top ten alignments in a PDB library. The unaligned regions of the sequence are built through ab initio folding, by considering the lowest free energy states and minimized sterical clashes that are identified by SPICKER and TM-align, respectively, and the final models are built at an atomic level by REMO which optimizes the hydrogen-bonding profile. In addition, the I-TASSER online software also allows the prediction of protein biological functions, as it compares the designed models to different libraries of proteins with previously identified functions [34, 35].

The docking analysis was performed using the software iGEMDOCK v. 2.1, a Generic Evolutionary Method for molecular DOCKing, developed by Jinn-Moon Yang (http://gemdock.life.nctu.edu.tw/dock/igemdock.php). It computes the interaction between one or more ligands with the active site of the target protein through empirical scoring function and an evolutionary approach algorithm. It also provides post-analysis tools by k-means and hierarchical clustering methods from the docked poses and the protein–ligand properties, such as atom composition and energy function [36–38].

To perform the docking analysis, structure of all the ligands was downloaded from the PubChem database (https://www.ncbi.nlm.nih.gov/pccompound) in MOL2 format. [lactose (CID 6134), maltose (CID 6255), mannose (CID 18950), xylose (CID 644160), cellobiose (CID 10712), and fructose (CID 5984)]. The PDB file for the model was uploaded to the iGEMDOCK software along with all the ligands, and the docking was performed using standard settings, with a population size of 200 and a total of 70 generations. The post-docking parameters were analyzed and the best docked pose for each ligand was visualized by The PyMOL Molecular Graphics System, Version 1.8.6.0 Schrödinger, LLC (https://pymol.org/2/).

## Results

### Structural characterization of the transporter *Tr69957*

**Fig. 1:**
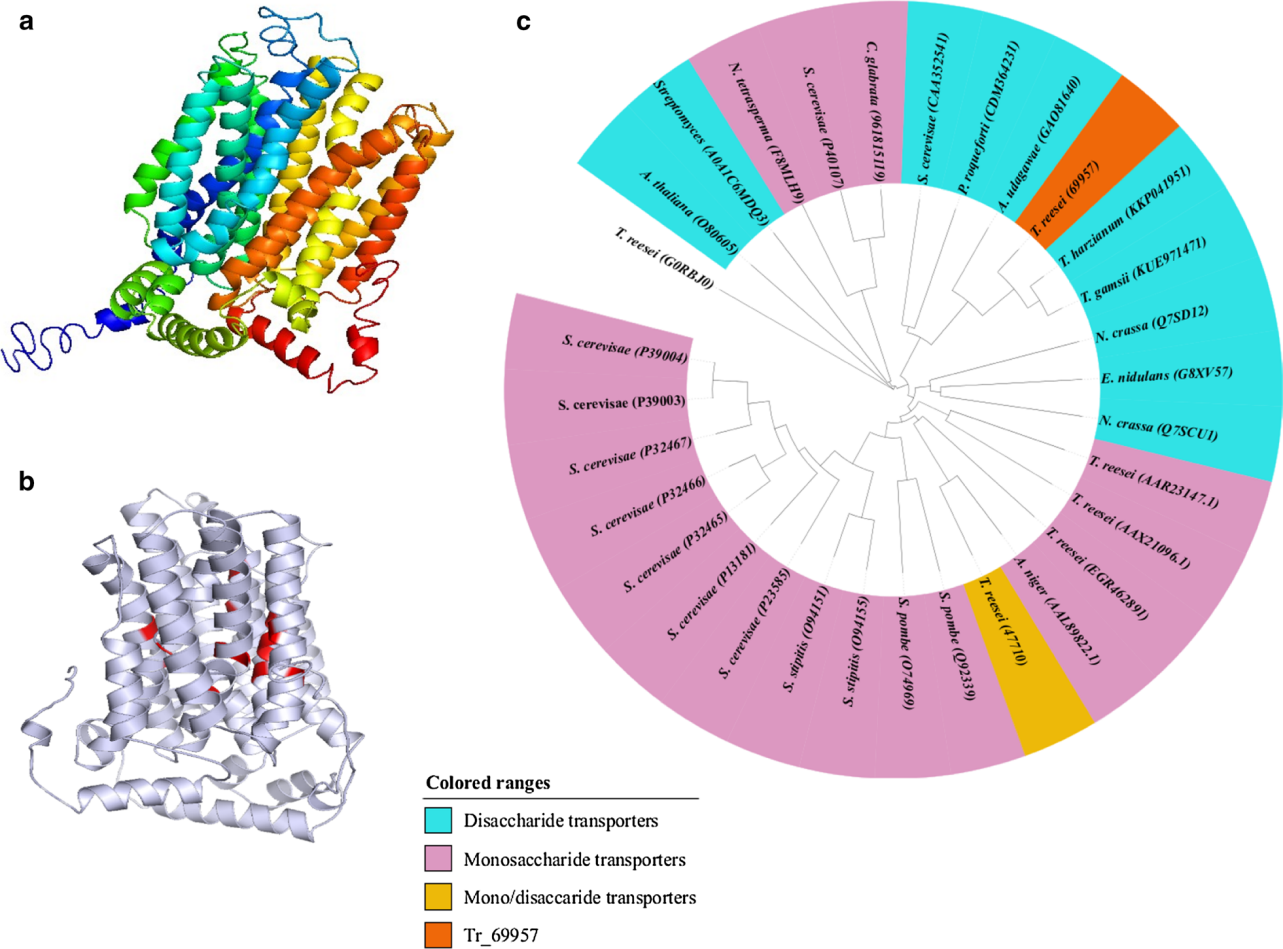
Structural analysis of *Tr69957*. **a** Three-dimensional structure of *Tr69957.* **b** Three-dimensional structure of *Tr69957*, structural motif (in red) conserved in other sugar transporters, which may be involved with the specificity for the transported sugar or attachment to the substrate. **c** Phylogenetic analysis of *Tr69957*. Sequence alignments were performed using the ClustalW program

The protein encoded by *Tr69957* is described as a maltose permease according to the genome database of *T. reesei*, available in the JGI Genome Portal (http://genome.jgi-psf.org/Trire2/Trire2.home.html). Considering all the parameters provided by the I-TASSER platform, the chosen model for the *Tr69957* (Fig. 1a) was predicted based on the template 4ZWB.A [human glucose transporter GLUT3 (N45T mutant) with bound d-glucose, outward-occluded conformation], which was determined by Deng et al. [39]. The predicted structure was considered to be adequate, as it presented a C-score of − 0.80 and a TM-score of 0.61 ± 0.14. It also presented a root mean square deviation (RMSD) of 9.3 ± 4.3, and the cluster density was of 0.0938 for 1483 decoys (Additional file 6). The local accuracy results for the selected model (normalized B-factor) can be found in the Additional file 7. The structural motif described for *Tr69957* (in red) (Fig. 1b) was conserved in several sugar transporters and may be involved with the specificity of the protein. The prediction of the *Tr69957* structure by the TMRPres2D tool suggests that this transporter has 12 transmembrane domains, with a large extracellular loop and a relatively long central loop that is shared by many sugar transporters (data not shown). Phylogenetic analysis of *Tr69957* with other fungal transporters indicated that *Tr69957* shows high homology with disaccharide transporters, including the maltose permeases of *T. harzianum*, *T. gamsii*, *Penicillium roqueforti,* and *S. cerevisiae* (Fig. 1c).

### *Tr69957* allows the growth of *S. cerevisiae* on various carbon sources

**Fig. 2:**
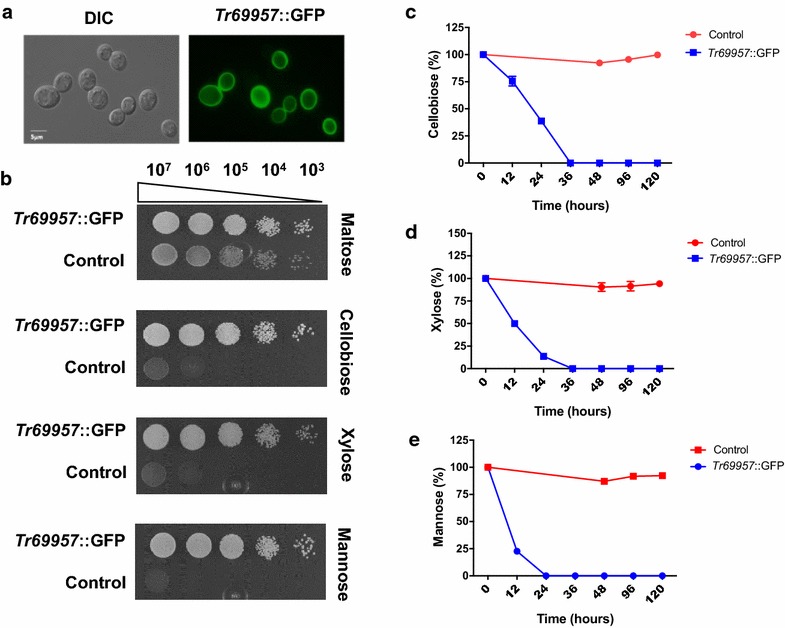
Effect of *Tr69957* transporter on growth of *S. cerevisiae* in the presence of cellobiose, mannose, and xylose. **a** *Tr69957*::GFP is localized in the plasma membrane in *S. cerevisiae* when grown in maltose-rich medium. Microscopy images were acquired in DIC and GFP fluorescence. **b** Growth of *S. cerevisiae* strain EBY.VW4000 transformed with (*Tr69957*) and without (control) the transporter *Tr69957* gene in the presence of 1% cellobiose, xylose, or mannose. Both the control and *Tr69957* strains contained the genes encoding enzymes for xylose and cellobiose metabolic pathways. Yeast strains were initially grown in maltose-rich medium before a serial dilution was done (1:10 dilution, initial optical density OD_600nm_ = 1.0) and cells were incubated on plates containing the different carbon sources at 30 °C for 96 h; **c** cellobiose, **d** xylose, and **e** mannose transport as determined by HPLC in the *S. cerevisiae Tr69957* strain in the presence of 1% of the specific carbon source. *S. cerevisiae* strain which did not contain the *Tr69957* transporter was used as the control strain. Standard deviations were obtained from triplicate assays

To characterize the transporter *Tr69957*, we performed an analysis in which an *S. cerevisiae* strain EBY.VW4000, which lacks 20 distinct sugar transporters (but expresses XK, XR, XDH, and β-glucosidase genes [32]), was co-transformed with the *Tr69957* fused to the GFP. Fluorescence was predominantly observed on the yeast cell surface, suggesting that *Tr69957* is located on the yeast plasma membrane (Fig. 2a). To verify the functionality of the *Tr69957* in the *S. cerevisiae* strain EBY.VW4000, the complementary strain carrying the *Tr69957* fused to the GFP was grown in the presence of maltose (positive control), cellobiose, xylose, and mannose. The drop-out assay clearly showed that the expression of *Tr69957* was able to restore the growth of EBY.VW4000 on cellobiose, mannose, and xylose, suggesting that *Tr69957* is able to transport these three different sugars (Fig. 2b). However, *Tr69957* was not able to transport sophorose as expected (data not shown). The consumption of cellobiose, mannose, and xylose by the *S. cerevisiae* EBY.VW4000 and complemented strain expressing the *Tr69957* fused to the GFP was also evaluated. Under the three analyzed conditions, the yeast that does not contain the *Tr69957* was unable to transport the three carbon sources after 5 days of growth.

In contrast, the expression of *Tr69957 completely* restored the consumption of cellobiose, xylose, and mannose after 24 h (mannose) and 36 h (cellobiose and xylose) of growth (Fig. 2c–e). Altogether, these results suggest that *Tr69957* can act as a monosaccharide as well as a disaccharide transporter in the yeast *S. cerevisiae*.

### Disruption of *Tr69957* impairs the growth of *T. reesei* in the presence of monosaccharides and disaccharides

*Trichoderma reesei* Δ*69957* strain was constructed by homologous recombination using QM6aΔ*tmus53*Δ*pyr4* as the parental strain, whose non-homologous end joining pathway was disrupted [14]. Southern blot analysis of *Eco*RV-digested chromosomal DNA (Additional file 3) using the terminator region of *Tr69957* gene as a probe resulted in expected results in parental (3391 bp) as well as *Δ69957* (6698 bp) mutant strains, respectively (Additional file 3).

**Fig. 3:**
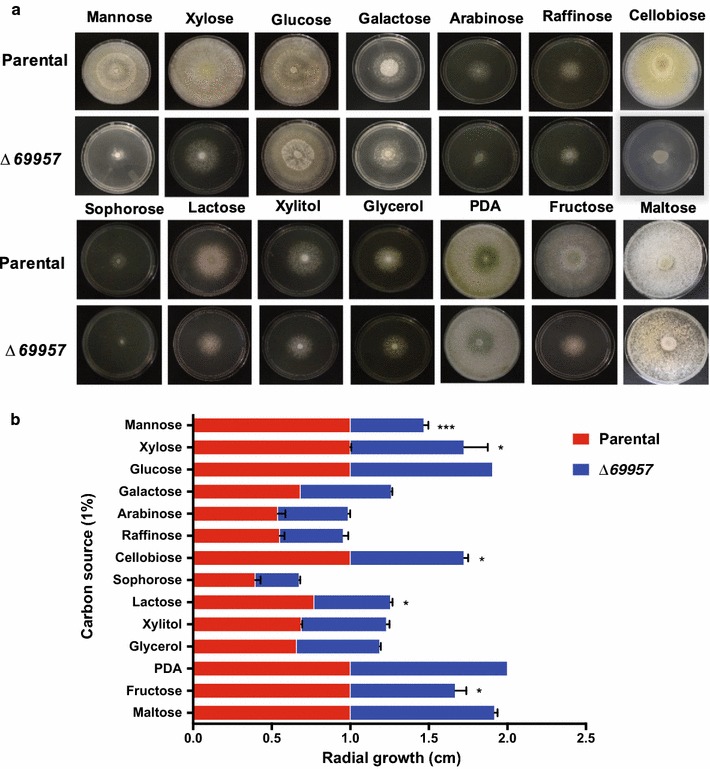
Growth of *T. reesei* parental strain and Δ*69957* strain on different carbon sources. **a** Evaluation of the growth of QM6a Δ*tmus53* Δ*Pyr4* CTC: *loxP* (parental) and Δ*69957* on different carbon sources. Images were acquired after 144 h of culture on MM plates supplemented with the specific carbon sources. **b** Evaluation of the growth halo of QM6a Δ*tmus53* Δ*Pyr4* CTC: *loxP* and Δ*69957* in different carbon sources. The growth halo of each line was analyzed in PDA medium, which was used as a positive control of growth of both lines. Standard deviations were obtained from triplicate assays. *p < 0.0001

After the disruption of *Tr69957* transporter, we evaluated the ability of the Δ*69957* mutant strain to grow in the presence of various carbon sources (Fig. 3). For this purpose, the parental and mutant strains were grown on MM plates in presence of d-glucose, d-fructose, d-mannose, d-(+) cellobiose, lactose, PDA, d-(+)-raffinose, l-arabinose, raffinose, glycerol, d-xylose, or xylitol as the sole carbon-source. The Δ*69957* mutant strain exhibited a decreasing radial growth in the presence of mannose, xylose, cellobiose, lactose, and fructose (Fig. 3a, b). Our results suggest that the transporter *69957* has a key role in the metabolism of monosaccharides such as mannose, xylose, and fructose, and disaccharides such as cellobiose and lactose, suggesting a promiscuous mechanism involved in the transport of sugar into the cell.

### *Tr69957* presents lower protein–ligand free energy in interactions involving disaccharides than that in the monosaccharides

To understand the preference of sugars transported by *Tr69957,* we performed a docking analysis. The selection of the most adequate model relies on the confidence scores: C-score and TM-score. The C-score estimates the quality of the prediction, for which the higher the value, the higher is the confidence. The TM-score latter measures the structural similarity between the predicted model and the template used for its construction. The values > 0.5 demonstrate a correct topology, whereas values < 0.17 demonstrate a random similarity. RMSD also represents the similarity of the predicted structure to the template, although it may show a very high value due to local errors, even if the model is adequate. The three scores are highly correlated to one another.

Considering all the parameters provided by the I-TASSER platform, the chosen model for the *Tr69957* was predicted based on the template 4ZWB.A [human glucose transporter GLUT3 (N45T mutant) with bound d-glucose, outward-occluded conformation], determined by Deng et al. [39]. The predicted structure was considered adequate, since it presented a C-score of − 0.80, TM-score of 0.61 ± 0.14, and an RMSD of 9.3 ± 4.3.

**Fig. 4:**
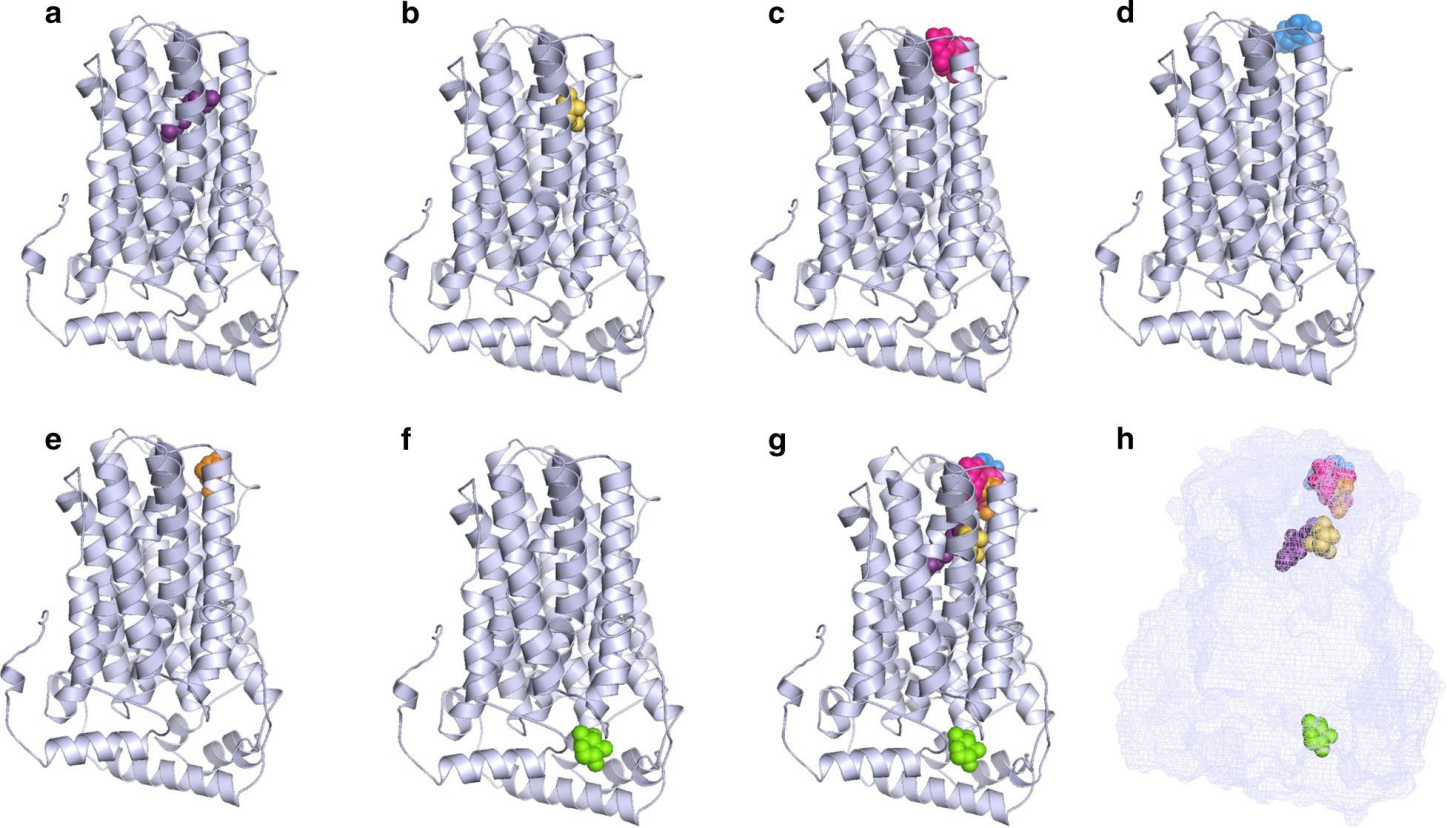
Docking analysis of the binding model of *Tr69957* with **a** cellobiose, **b** fructose, **c** lactose, **d** maltose, **e** mannose, **f** xylose, and all sugars together (**g**, **h**) was performed using the iGEMDOCK v2.1 program

The PDB file obtained for the *Tr69957* was used for the docking analysis and the results were post-analyzed based on the total free energy, as well as the Van der Waals, hydrogen, and electrostatic interactions (Table 1) for each ligand. Lactose is the carbohydrate with the most favorable binding (Fig. 4c) and xylose (Fig. 4f) with the most unfavorable binding (Fig. 4) when the rigid structure of the protein, but with the adequate ligand flexibility was considered. The complete interaction profiles can be found in the Additional file 8.

**Table 1:** Tr_69957 interactions with different sugars analyzed by docking based on the total free energy, the Van der Waals, hydrogen, and electrostatic interaction for each ligand

Interaction	Energy (KJ/mol)	VDW	H bonds
Tr69957::cellobiose	− 108.48	− 66.3	− 44.8
Tr69957::fructose	− 81.47	− 52.9	− 28.5
Tr69957::maltose	− 101.8	− 59.3	− 42.5
Tr69957::mannose	− 80.34	− 37.18	− 43.15
Tr69957::xylose	− 77.12	− 27.8	− 49.3
Tr69957::lactose	− 114.71	− 83.05	− 31.6

These results show that the *Tr69957* sugar transporter is able to transport several types of carbohydrates, preferentially disaccharides, such as lactose, cellobiose, and maltose in which the molecules showed the lowest free energies, and thus, the highest binding affinity to the protein (Table 1). With respect to the monosaccharides, fructose (Fig. 4b), mannose (Fig. 4e), and xylose (Fig. 4f), even though these exhibited higher free energy than the disaccharides, they still possess adequate binding affinity, suggesting that the conformational changes the protein may suffer are adaptive to the distinct carbon sources in the environment (Table 1).

### Disruption of *Tr69957* Impairs the Fungal Biomass Accumulation and the Sugar Uptake

The early gene expression experiments performed to characterize the *Tr69957* sugar transporter were done using SCB, a complex lignocellulosic biomass. Similar to other plant cell walls, SCB is mainly formed by two carbohydrate fractions (cellulose and hemicellulose) embedded in a lignin matrix. The hydrolysis of SCB releases a range of sugars, such as mannose, xylose, cellobiose, and fructose that can be used by the fungus for energy production. As demonstrated in Fig. 3a, b, the Δ*69957* mutant strain showed an impaired growth in the presence of all these sugars released from SCB hydrolysis. In this way, for the next set of experiments for characterization of *Tr69957*, we selected three out of the four sugars that presented significant variations during the growth conditions of the mutant strain.

**Fig. 5:**
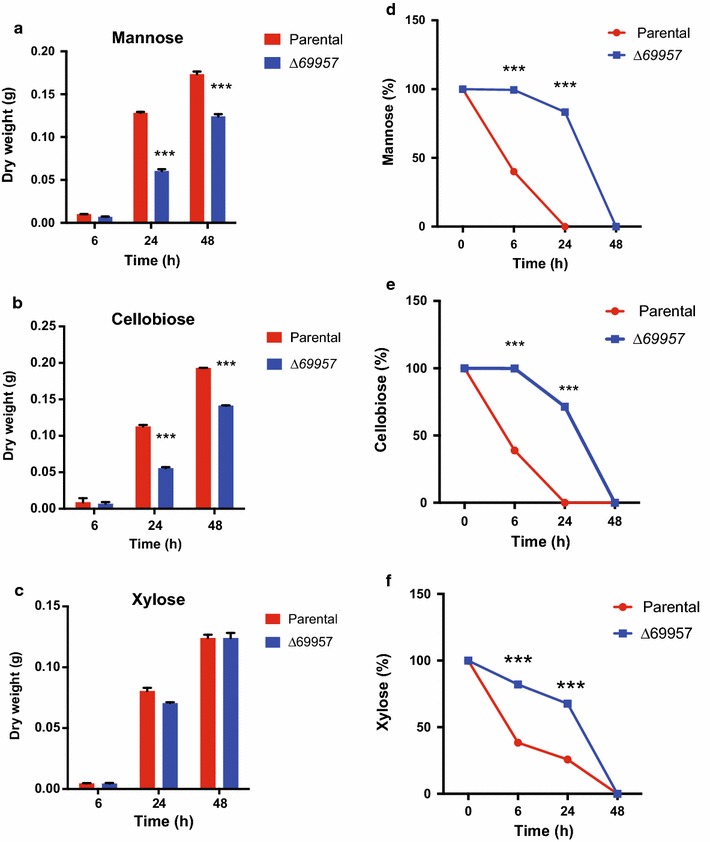
*Tr69957* disruption decreases the growth and consumption of mannose, cellobiose and xylose in *T. reesei* strain. Analysis of the biomass and sugar consumption of parental and mutant strains under culture in MAM supplemented with mannose (**a**, **d**), cellobiose (**b**, **e**), and xylose (**c**, **f**), during growth for 0, 6, 24, and 48 h in the presence of 1% mannose, cellobiose, or xylose. Standard deviations were obtained from triplicate assays. *p < 0.0001

The deletion of the sugar transporter-encoding *Tr69957* resulted in reduced fungal biomass accumulation in the presence of mannose (Fig. 5a) and cellobiose (Fig. 5b), which does not occur in the presence of xylose (Fig. 5c). Interestingly, our results showed that the deletion of sugar transporter *Tr69957* altered the uptake kinetics of mannose, cellobiose, and xylose in *T. reesei* in comparison with that of *S. cerevisiae.* In regards to mannose, we identified that this sugar is completely consumed by the parental strain after 24 h of cultivation (Fig. 5d). Conversely, in the Δ*69957,* this monosaccharide was only partially consumed after 48 h of cultivation (Fig. 5d). The same profile was observed for the uptake kinetics of cellobiose (Fig. 5e). However, we observed that xylose uptake showed a slight difference in the mutant strain as compared to the parental strain. Our results showed that the xylose content was completely exhausted in parental and mutant strain only after 48 h of cultivation (Fig. 5f). In addition, we also observed that uptake kinetics for all the sugars was faster in the parental strain as compared to that in the Δ*69957* mutant strain, which showed a delay in the sugar consumption (Fig. 5c, d, f). Together, these results suggest that there is a delay in the attainment of mannose, cellobiose, and xylose in the mutant strain. In addition, our results showed that *Tr69957* is important for the recognition and transport of mannose, cellobiose, and xylose, and probably, different mechanisms could control the uptake of these sugars in *T. reesei*.

**Fig. 6:**
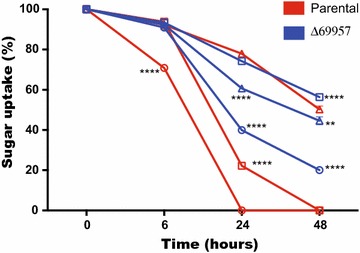
Rate of sugars consumption by the parental and mutant strains during culture in MAM supplemented with mannose (white circle), xylose (white square), and cellobiose (white triangle),for 0, 6, 24, and 48 h growth in the presence of 30 mM of each sugar. Standard deviations were obtained from triplicate assays. *p < 0.0001

To test this hypothesis, we evaluated the consumption of these sugars during the cultivation by the addition of the three sugars in the same culture (Fig. 6). The results demonstrated that mannose is the first sugar to be consumed in the parental strain, followed by xylose and cellobiose. In the Δ*69957* mutant strain, we observed a distinct profile of sugar consumption, mannose being the first sugar consumed after 48 h of cultivation followed by cellobiose and xylose. In this way, our results showed a delay in the consumption of xylose in the mutant strain as compared to the parental strain. Furthermore, we also observed that at 6 h, the mutant strain cannot consume mannose in a similar manner to that by the parental strain. After 24 h, approximately 60% of the sugars were consumed by the mutant strain, while in the parental strain, all the sugars present in the medium had already been consumed (Fig. 6). However, the profile of the cellobiose consumption appeared to be different with respect to the parental and mutant strains when compared to the other carbon sources that were analyzed. The mutant strain *69957* presented a cellobiose consumption about 1.3 times higher than the parental strain after 24 h of culture, whereas this rate persisted until 48 h (Fig. 6), suggesting that the more rapid consumption of cellobiose may well result from the increased hydrolysis by β-glucosidases in the absence of *Tr69957.*

### Tr69957 affects cellulolytic and hemicellulolytic gene expression in *T. reesei*

**Fig. 7:**
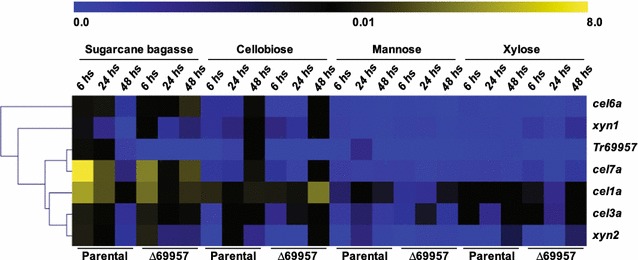
*Tr69957* affects cellulolytic and hemicellulolytic gene expression in *T. reesei* during the growth in different carbon sources. Analysis of the expression of *cel6a*, *xyn1*, *Tr69957*, *cel7a*, *cel1a*, *cel3a*, and *xyn2* during cultivation of the mutant and parental strains grown in MAM supplemented with cellobiose, mannose, xylose, or SCB as the sole carbon source. Evaluation of cellulase gene expression was calculated according to the 2^−ΔΔCT^ method (Livak and Schmittgen [31])

Since the deletion of *Tr69957* affected the fungal growth and sugar consumption, we evaluated the gene expression profile of six cellulases and hemicellulases in the presence of SCB, cellobiose, mannose, and xylose during 6, 24, and 48 h of incubation of the parental and mutant strains. In addition, the expression of *Tr69957* was also evaluated (Fig. 7).

After 48 h of growth, the highest expression of *Tr69957* was achieved in the presence of cellobiose. Under similar conditions, deletion of *Tr69957* resulted in a drastic decrease in the expression of *cel7a*, *cel6a,* and *cel3a* in the mutant strain, whereas *cel1a* and *xyn2* were less expressed in the parental QM6a (Fig. 7). In the presence of mannose and xylose, the transporter *Tr69957* was less expressed in comparison with that in the other conditions. In both carbon sources, the *cel1a* was the most affected gene with the *Tr69957* deletion, being more expressed in the parental QM6a when compared to the mutant strain after 24 h of cultivation (Fig. 7).

The growth in SCB showed the highest expression of *Tr69957* among all the tested conditions, with maximum expression at 6 h of growth. In SCB, the expression of *cel7a* and *cel6a* was decreased in the mutant Δ*69957* in comparison with the parental strain in the first few hours of growth. Interestingly, after 48 h, the deletion of *Tr69957* resulted in increased expression of all the cellulolytic and hemicellulolytic genes analyzed in the mutant strain (Fig. 7). Figure 7 shows that *cel1a* and *cel3a* were significantly induced in *∆69957* in the presence of sugarcane bagasse after 24 and 48 h when compared to the WT strain in the same conditions. This result is in accordance with the assumption that the absence of *Tr69957* promotes faster consumption of cellobiose due to the increased hydrolysis by β-glucosidases in the presence of mixture of carbon sources like mannose, cellobiose, and xylose, which also compose the sugarcane bagasse.

## Discussion

The fungus *T. reesei*, in the absence of simple sugars as carbon sources, secretes extracellular lignocellulolytic enzymes, which can degrade biomass through a natural process [1, 40]. Thereafter, the degraded simple sugars can be assimilated for cell metabolism. In this process, transporters are the key role players in *T. reesei* to sense and utilize sugars [7, 29]. Most transporters encoded by filamentous fungi have not yet been characterized. Therefore, studying the function of transporters will provide new insights to understand the sensing mechanism in *T. reesei*. This study will enable us to better understand the gene expression pattern of cellulolytic enzymes produced by this fungus, which can contribute to the performance of it in the bioethanol industry. In this study, we characterized a putative sugar transporter *Tr69957* that was previously identified by our group using RNA-seq data, which is important for xylose, cellobiose, and mannose utilization in *T. reesei*. To the best of our knowledge, this is the first report of a potential mannose transporter and its involvement in the degradation process of cellulose in the filamentous fungus *T. reesei*.

For better demonstration of the phenomenon that *Tr69957* is able to transport specific sugars, the *S. cerevisiae* strain EBY.VW4000 [13] was used. The *S. cerevisiae* strain (EBY.VW4000) lacks the hexose transport activity due to the deletion of 20 hexose transporter genes and is unable to grow on glucose, fructose, mannose, or galactose as the sole carbon source. The hexose transport-deficient *S. cerevisiae* strain (EBY.VW4000) has been an important tool for characterizing new hexose transporters of other fungi, such as HXTB [41] and RhtA [42] from *A. niger,* and the TBHXT1 [43] transporter from the ascomycete *Tuber borchii*. Using this *S. cerevisiae* strain, we can prevent interference from other sugar transporters that were already described in *T. reesei*. The yeast experiments suggest that *Tr69957* has the capability of transporting a few sugars, including cellobiose, mannose, and xylose. Therefore, *Tr69957* can transport more than one sugar, which is not surprising because the filamentous fungi are able to internalize a wide variety of mono- and oligosaccharides and it has already been shown that transporters expressed by them can often transport more than one type of sugar [8].

Our data showed that *Tr69957* probably participated in the transport of cellobiose, mannose, xylose, lactose, galactose, and fructose in *T. reesei*, as *△Tr69957* strain displayed growth defects on these carbon sources. However, the docking results showed that *Tr69957* exhibits lower protein-binding energy in interactions involving disaccharides compared to the monosaccharides, suggesting a better orientation and affinity between these molecules.

The absence of transporter specificity can be explained by the similarity in the structures of some of the sugar molecules. Furthermore, some of these proteins can act as transporters as well as nutrient sensors [44]. Substrate transport requires binding and subsequent conformational change. After binding of the substrate, MFS transporters go through a series of conformational changes [45]. Many residues in MFS transport structures are involved in substrate uptake [46]. Besides substrate binding and specific conformational changes, the transporter domains function as gates that influence the substrate specificity [47]. Structurally, similar substrates can bind to the transporter; however, they cannot induce the conformational change, leading to inhibition of transport; for example, d-glucose can competitively inhibit d-xylose transporters as they share similarity in their structures [48]. However, interesting exceptions have been discovered in few *A. niger* sugar transporters, which suggest that if the binding occurs without subsequent conformational change, then the alternative substrate will be transported across the membrane [49].

Conversely, the absence of specificity can be advantageous when fungi encounters complex lignocellulosic material, which contains multiple hexoses and pentoses [11]. The fungi can exhibit differences and similarities in response to different lignocellulosic substrates [1, 50, 51].

**Fig. 8:**
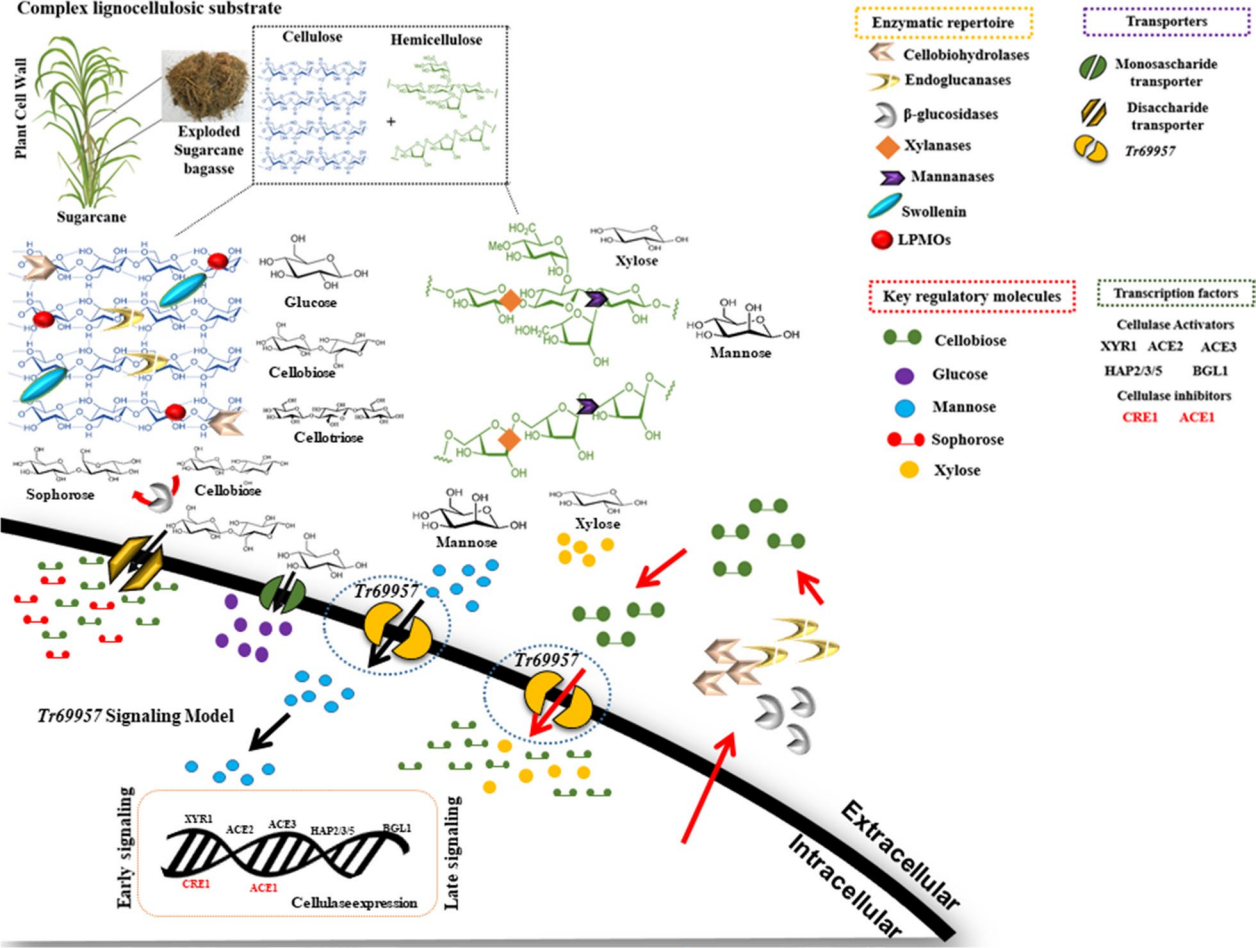
Hypothetical model of *Tr69957*-mediated cellulase induction in *T. reesei*. The figure shows the potential mechanism of signaling mediated by the sugar transporter *Tr69957* under the control of cellulase expression. This scheme also shows the processes involved in regulating lignocellulosic enzymes in *T. reesei*. The major enzymes involved in mobilization of lignocellulosic material are endoglucanases (EGs), cellobiohydrolases (CBHs), and β-glucosidases. These enzymes act synergistically to break down biomass. Accessory enzymes such as swollenins and Lytic Polysaccharide Monooxygenases (LPMOs) are also important in this process. Due to its complexity, the degradation of lignocellulosic material involves other enzymes such as xylanases and mannanases that are able to break down the polysaccharides present in the plant biomass. The expression of all the degrading enzymes is controlled by the major transcription factors (TFs): XYR1, ACE1, ACE2, ACE3, BGL1, CRE1, and the HAP2/3/5 complex. These transcription factors can also regulate the expression of sugar transporters that are responsible for sugar uptake in response to different carbon sources. Early and late signaling involving the sugar transporter *Tr69957* are indicated by black and red arrows, respectively

In Fig. 6, we observed that when a mixture of mannose, cellobiose, and xylose is present, the parental strain consumes in the order of mannose, xylose, and cellobiose. Despite the molecular docking results showing that *Tr69957* exhibits more affinity towards cellobiose than towards mannose and xylose, the presence of these sugars can cause reduction of cellobiose uptake. Similar results were observed in MstG and MstH *A. niger* sugar transporters. Both transporters showed high specificity for glucose. However, some sugars like galactose, fructose, and xylose were able to inhibit MstG glucose transport by 39, 29, and 25%, respectively; in the MstH transformants, the presence of mannose and fructose reduced the uptake of labeled glucose by 70 and 50%, respectively [52]. Moreover, in *A. niger*, substrate specificity of hexose transporters HxtB and HxtC showed a reduction in glucose uptake by 70–80% in presence of galactose, fructose, and mannose [48]. In the mutant strain, we can observe a different pattern of sugar consumption, where mannose is consumed first, then cellobiose and xylose at last, in addition to having higher cellobiose consumption than the parental strain under the similar conditions. Similar results were found in case of STP1, a *T. reesei* cellobiose transporter mutant strain. In comparison with the WT, cellobiose in the culture supernatant of STP1 mutant was consumed at a much faster rate [8]. However, the same does not occur when we culture the Δ*69957* mutant strain containing only cellobiose as the sole source of carbon (Fig. 4d). We believe that in the presence of these three sugars or in complex carbon sources, for example, lignocellulosic material, a specific response occurs in the absence of *Δ69957*, although the exact mechanism awaits further elucidation. We suppose that, in the presence of *Tr69957*, mannose and xylose, which are present in the hemicellulosic portion, somehow signal the presence of cellulose in the environment when transported at first. This transporter could be dependent on an early and late signaling mechanism in response to lignocellulosic biomass. The hemicellulose polymer includes xylan, glucuronoxylan, arabinoxylan, glucomannan, and xyloglucan. In addition, these polysaccharides contain many different sugar monomers such as xylose, arabinose, mannose, glucose, galactose, and rhamnose, being xylose the most abundant one in most of the cases, although sometimes, mannose can be the most abundant sugar [53, 54] (Fig. 8). The polysaccharides found in the hemicellulose structure can be hydrolyzed by xylanases and mannanases releasing xylose and mannose, respectively, that in turn could be transported into the cell. In our model, we believe that *Tr69957* may sense the presence of mannose in the extracellular compartment and the transport of mannose into intracellular milieu would in turn activate the cellulases expression in an early signaling involving this sugar transport (Fig. 8—black arrows). Here, we consider that *Tr69957* can act as a potential mannose transporter, which could be involved in the degradation process of cellulose in *T. reesei*.

Concomitantly, in a second instance, in response to early signaling, *Tr69957*-mediated cellulase expression and secretion of CAZymes could act by cleaving cellulose to release disaccharides, such as cellobiose, that in turn might get internalized promoting a late signaling response, increasing the expression of cellulases in the presence of cellulose (Fig. 8—red arrows). Furthermore, as observed in Fig. 7, in the presence of mannose, the expression of β-glucosidase is higher than that of other cellulases, indicating that mannose present in the environment might signal the expression of these enzymes, which act in the conversion of cellobiose to sophorose [55], thereby increasing the cellulase expression during the late signaling of *Tr69957*.

In this study, we characterized a cellobiose, mannose, and xylose transporter involved in SCB degradation in *T. reesei*. The study also suggests that the transporter is associated with early and late signaling in the presence of lignocellulosic biomass. Taken together, these data contribute to the studies addressing the mechanism of action by which *T. reesei* senses the cellulose signal. The present study explores the mechanism of sensing and transportation of sugars in *T. reesei*, which are important and relevant during the application of this fungus in bioethanol production.

## Conclusion

The present study aimed to improve our understanding of the sugar transport systems, through characterization of a novel sugar transporter involved in the degradation of lignocellulosic biomass in *T. reesei.* In addition, this study will aid in understanding the influence of this transporter on metabolic signaling and induction of the cellulase genes, which will subsequently have effect on lignocellulose degradation.

Our results showed that the transporter *Tr69957* of *T. reesei* can transport cellobiose, xylose, and mannose, and can affect the expression of a few genes encoding enzymes, such as cellulases and xylanases, in the presence of SCB. Moreover, our study is the first report that shows a potential role for a mannose transporter in the regulation of cellulase genes in *T. reesei* in the presence of SCB. These results suggest that *Tr69957* can be important during the signaling of cellulase gene expression. These findings contribute to the understanding of the molecular mechanisms involved in the regulation of the processes of cellulolytic enzyme synthesis and signaling.

## Additional files

**Additional file 1.** Strains and plasmids used in this work.

**Additional file 2.** Oligonucleotides used for *pyrG* amplification, promoter region and terminator region of *69957*.

**Additional file 3.** Southern blot analyse to confirm deletion of *Tr69957* in *T. reesei*. (A) Strategy used to confirm deletion of *Tr69957* by Southern blot. The figure shows the promoter region (in green) and terminator (in purple) of the gene, with the substitution of the ORF by the *pyrG* selection marker. A specific DNA probe was created to bind the portion of the gene (in blue), confirming its presence in the analyzed genetic content. (B) Southern blot showing the parental fungus (QM6aΔ*tmus53*Δ*pyr4*) used as control and four deleted candidates that did not present detection of the gene portion equivalent to 3391 bp as in the parental and presented the region corresponding to 6698bp, confirming the deletion.

**Additional file 4.** Oligonucleotides used for evaluation of gene expression.

**Additional file 5.** Oligonucleotides used for complementation in *S. cerevisiae*.

**Additional file 6.** SCORING FUNCTIONS: the global accuracy for the five designed candidates. The chosen structure was the Model 1, which was predicted based on the 4ZWB.A (human GLUT3 glucose transporter (N45T mutant) with bound d-glucose, outward-occluded conformation), determined by Deng et al. [39], since it presents not only the highest value for C-score, but also satisfactory values for the TM-score and the RMSD, also being the largest cluster. C-score: the confidence of the construction essentially based on the template alignments and the parameters for assembling the structure, whereas the higher the value, the higher the confidence and quality. TM-Score and RMSD: the similarity between the designed model and the template used, being less and more sensitive to local error, respectively. TM-score > 0.5 indicates a correct topology. Number of decoys: the amount of conformations generated by the software. Cluster density: the size of the clusters formed for each model, whereas the larger the cluster, the lowest the free energy of the model.

**Additional file 7.** LOCAL ACCURACY: the divergence of the modeling represented by the distance (Å) between the protein designed and the template used for the construction of it. RES: residue number; SS: predicted secondary structure (C: random coil, H: alpha-helix, S: beta-strand; SA: predicted solvent accessibility at 25% cutoff (E: exposed, B: buried); COV: alignment coverage; BFP: predicted normalized B-factor; RSQ_1: Residue-Specific Quality of the template (the estimated deviation of the residue on the template from the built protein)).

**Additional file 8.** INTERACTION PROFILE: the detailed amount of energy required for each atom in the protein to interact with each one of the ligands, through hydrogen bonding (H) and Van der Waals contact (V).

## References

[1] Daly P, van Munster JM, Raulo R, Archer DB, Silva RN (2016). Transcriptional regulation and responses in filamentous fungi exposed to lignocellulose. Fungal biotechnology for biofuel production.

[2] Martinez D, Berka RM, Henrissat B, Saloheimo M, Arvas M, Baker SE (2008). Genome sequencing and analysis of the biomass-degrading fungus *Trichoderma reesei* (syn. *Hypocrea jecorina*). Nat Biotechnol.

[3] Castro LDS, Antoniêto ACC, Pedersoli WR, Silva-Rocha R, Persinoti GF, Silva RN (2014). Expression pattern of cellulolytic and xylanolytic genes regulated by transcriptional factors XYR1 and CRE1 are affected by carbon source in *Trichoderma reesei*. Gene Expr Patterns.

[4] Häkkinen M, Arvas M, Oja M, Aro N, Penttilä M, Saloheimo M (2012). Re-annotation of the CAZy genes of *Trichoderma reesei* and transcription in the presence of lignocellulosic substrates. Microb Cell Fact.

[5] Gusakov AV (2011). Alternatives to *Trichoderma reesei* in biofuel production. Trends Biotechnol.

[6] Ragauskas AJ, Williams CK, Davison BH, Britovsek G, Cairney J, Eckert CA (2006). The path forward for biofuels and biomaterials. Science.

[7] Sloothaak J, Antonio J, Ramos T, Odoni DI, Laothanachareon T, Derntl C (2016). Biotechnology for Biofuels Identification and functional characterization of novel xylose transporters from the cell factories *Aspergillus niger* and *Trichoderma reesei*. Biotechnol Biofuels.

[8] Zhang W, Kou Y, Xu J, Cao Y, Zhao G, Shao J (2013). Two major facilitator superfamily sugar transporters from *Trichoderma reesei* and their roles in induction of cellulase biosynthesis. J Biol Chem.

[9] Ries L, Pullan ST, Delmas S, Malla S, Blythe MJ, Archer DB (2013). Genome-wide transcriptional response of *Trichoderma reesei* to lignocellulose using RNA sequencing and comparison with *Aspergillus niger*. BMC Genom.

[10] Fernanda T, Borborema P, De Lima A, Parachin NS, Mingossi FB, Velasco J (2016). Biotechnology for biofuels identification and characterization of putative xylose and cellobiose transporters in *Aspergillus nidulans*. Biotechnol Biofuels.

[11] Colabardini AC, Nicolas L, Ries A, Brown NA, Fernanda T, Savoldi M (2014). Functional characterization of a xylose transporter in *Aspergillus nidulans*. Biotechnol Biofuels.

[12] Zhang W, Kou Y, Xu J, Zhao G, Shao J, Wang H (2013). Microbiology: two major facilitator superfamily sugar transporters from *Trichoderma reesei* and their roles in induction of cellulase biosynthesis. J Biol Chem.

[13] Wieczorke R, Krampe S, Weierstall T, Freidel K, Hollenberg CP, Boles E (1999). Concurrent knock-out of at least 20 transporter genes is required to block uptake of hexoses in *Saccharomyces cerevisiae*. FEBS Lett.

[14] Derntl C, Kiesenhofer DP, Mach RL, Mach-Aigner AR (2015). Novel strategies for genomic manipulation of *Trichoderma reesei* with the purpose of strain engineering. Appl Environ Microbiol.

[15] Schmoll M, Schuster A, Silva RDN, Kubicek CP (2009). The G-alpha protein GNA3 of *Hypocrea jecorina* (anamorph *Trichoderma reesei*) regulates cellulase gene expression in the presence of light. Eukaryot Cell.

[16] Souza D, De Souza WR, De Gouvea PF, Savoldi M, Bernardes LADS (2011). Transcriptome analysis of *Aspergillus niger* grown on sugarcane bagasse. Biotechnol Biofuels.

[17] Miller GL (1959). Use of dinitrosaiicyiic acid reagent for determination of reducing sugar. Anal Chem.

[18] Schuster A, Bruno KS, Collett JR, Baker SE, Seiboth B, Kubicek CP (2012). A versatile toolkit for high throughput functional genomics with *Trichoderma reesei*. Biotechnol Biofuels.

[19] Christianson TW, Sikorski RS, Dante M, Shero JH, Hieter P (1992). Multifunctional yeast high-copy-number shuttle vectors. Gene.

[20] Loprete D (2007). The protein kinase C orthologue PkcA plays a role in cell wall integrity and polarized growth in *Aspergillus nidulans* The protein kinase C orthologue PkcA plays a role in cell wall integrity and polarized growth in *Aspergillus nidulans*. Fungal Genet Biol.

[21] Colot HV, Park G, Turner GE, Ringelberg C, Crew CM, Litvinkova L (2006). A high-throughput gene knockout procedure for *Neurospora* reveals functions for multiple transcription factors. PNAS.

[22] Construction H, Deletion G, Collopy PD, Colot HV, Park G, Ringelberg C (2010). High-throughput construction of gene deletion cassettes for generation of *Neurospora crassa* knockout strains. Methods Mol Biol.

[23] Gietz RD, Schiestl RH (2008). High-efficiency yeast transformation using the LiAc/SS carrier DNA/PEG method. Nat Protoc.

[24] Gietz RD, Schiestl RH (1991). Applications of high efficiency lithium acetate transformation of intact yeast cells using single-stranded nucleic acids as carrier. Yeast.

[25] Goldman GH, Marques R, Luciano A, Bernardes DS, Quiapin C, Vitorelli PM (2003). Expressed sequence tag analysis of the human pathogen *Paracoccidioides brasiliensis* yeast phase: identification of putative homologues of *Candida albicans* virulence and pathogenicity genes. Eukaryot Cell.

[26] Sambrook J, Russell DW, Sambrook J, David W (2001). Preparation of cDNA libraries and gene identification. Molecular cloning a laboratory manual.

[27] Cardoza RE, Malmierca MG, Hermosa MR, Alexander NJ, Mccormick SP, Proctor RH (2011). Identification of loci and functional characterization of *Trichothecene* biosynthesis genes in filamentous fungi of the genus *Trichoderma*. Appl Environ Microbiol.

[28] Vizcaı JA, Gonza F, Elena R, Llobell A, Monte E, Gutie S (2007). Partial silencing of a hydroxy-methylglutaryl-CoA reductase-encoding gene in *Trichoderma harzianum* CECT 2413 results in a lower level of resistance to lovastatin and lower antifungal activity. Fungal Genet Biol.

[29] Huang Z, Chen X, Qin L, Wu H, Su X (2015). A novel major facilitator transporter TrSTR1 is essential for pentose utilization and involved in xylanase induction in *Trichoderma reesei*. Biochem Biophys Res Commun.

[30] Verbeke J, Coutinho P, Mathis H, Quenot A, Record E, Asther M (2009). Transcriptional profiling of cellulase and expansin-related genes in a hypercellulolytic *Trichoderma reesei*. Biotechnol Lett.

[31] Livak KJ, Schmittgen TD (2001). Analysis of relative gene expression data using real-time quantitative PCR and the 2−ΔΔCT method. Methods.

[32] Galazka JM, Tian C, Beeson WT, Martinez B, Glass NL, Cate JHD (2010). Cellodextrin transport in yeast for improved biofuel production. Science.

[33] Schiestl RH, Gietz RD (1989). High efficiency transformation of intact yeast cells using single stranded nucleic acids as a carrier. Curr Genet.

[34] Yang J, Yan R, Roy A, Xu D, Poisson J, Zhang Y (2014). The I-TASSER Suite: protein structure and function prediction. Nat Methods.

[35] Zhang Y (2008). I-TASSER server for protein 3D structure prediction. BMC Bioinform.

[36] Yang JM, Chen CC (2004). GEMDOCK: a Generic Evolutionary Method for Molecular Docking. Proteins Struct Funct Genet.

[37] Yang JM, Frank Hsu D (2005). Consensus scoring criteria in structure-based virtual screening. Emerg Inf Technol Conf.

[38] Yang JM (2004). Development and evaluation of a generic evolutionary method for protein-ligand docking. J Comput Chem.

[39] Deng D, Sun P, Yan C, Ke M, Jiang X, Xiong L (2015). Molecular basis of ligand recognition and transport by glucose transporters. Nature..

[40] Znameroski EA, Glass NL (2013). Using a model filamentous fungus to unravel mechanisms of lignocellulose deconstruction. Biotechnol Biofuels.

[41] Dos Reis TF, Menino JF, Bom VLP, Brown NA, Colabardini AC, Savoldi M (2013). Identification of glucose transporters in *Aspergillus nidulans*. PLoS ONE.

[42] Sloothaak J, Odoni DI, Martins dos Santos VAP, Schaap PJ, Tamayo-Ramos JA (2016). Identification of a novel l-rhamnose uptake transporter in the filamentous fungus *Aspergillus niger*. PLoS Genet.

[43] Polidori E, Ceccaroli P, Saltarelli R, Guescini M, Menotta M, Agostini D (2007). Hexose uptake in the plant symbiotic ascomycete *Tuber borchii* Vittadini: biochemical features and expression pattern of the transporter TBHXT1. Fungal Genet Biol.

[44] dos Reis TF, de Lima PBA, Parachin NS, Mingossi FB, de Castro Oliveira JV, Ries LNA (2016). Identification and characterization of putative xylose and cellobiose transporters in *Aspergillus nidulans*. Biotechnol Biofuels.

[45] Quistgaard EM, Löw C, Guettou F, Nordlund P (2016). Understanding transport by the major facilitator superfamily (MFS): structures pave the way. Nat Rev Mol Cell Biol.

[46] Zhang W, Cao Y, Gong J, Bao X, Chen G, Liu W (2015). Identification of residues important for substrate uptake in a glucose transporter from the filamentous fungus *Trichoderma reesei*. Sci Rep.

[47] Diallinas G (2014). Understanding transporter specificity and the discrete appearance of channel-like gating domains in transporters. Front Pharmacol.

[48] Farwick A, Bruder S, Schadeweg V, Oreb M, Boles E (2014). Engineering of yeast hexose transporters to transport d-xylose without inhibition by d-glucose. Proc Natl Acad Sci.

[49] Sloothaak J. Chapter 7: general discussion. In: the uptake of carbon sources by *Aspergillus niger*. 2017. http://library.wur.nl/WebQuery/wurpubs/fulltext/414746. Accessed 27 Sept 2017.

[50] Tian C, Beeson WT, Iavarone AT, Sun J, Marletta MA, Cate JHD (2009). Systems analysis of plant cell wall degradation by the model filamentous fungus *Neurospora crassa*. Proc Natl Acad Sci.

[51] Pribowo A, Arantes V, Saddler JN (2012). The adsorption and enzyme activity profiles of specific *Trichoderma reesei* cellulase/xylanase components when hydrolyzing steam pretreated corn stover. Enzyme Microb Technol.

[52] Sloothaak J, Odoni DI, de Graaff LH, Dos Santos VM, Schaap PJ, Tamayo-Ramos JA (2015). *Aspergillus niger* membrane-associated proteome analysis for the identification of glucose transporters. Biotechnol Biofuels.

[53] Gibson LJ (2015). The hierarchical structure and mechanics of plant materials. J R Soc Interface.

[54] Scheller HV, Ulvskov P (2010). Hemicelluloses. Annu Rev Plant Biol.

[55] Dos Santos Castro L, Pedersoli WR, Antoniêto ACC, Steindorff AS, Silva-Rocha R, Martinez-Rossi NM (2014). Comparative metabolism of cellulose, sophorose and glucose in *Trichoderma reesei* using high-throughput genomic and proteomic analyses. Biotechnol Biofuels.

