# Supplementary material for: Characterization of a novel sugar transporter involved in sugarcane bagasse degradation in Trichoderma reesei
Source: Biotechnol Biofuels. 2018 Apr 2;11:84. doi: 10.1186/s13068-018-1084-1 (PMC5879799; doi:10.1186/s13068-018-1084-1)
Supplement: Supplementary file 1 — Additional file 1. Strains and plasmids used in this work. [file 13068_2018_1084_MOESM1_ESM.pdf]

Additional file 1. Strains and plasmids used in this work.

Strains/plasmids	Genotype
S. cerevisae	
BY.VW4000	CEN.PK2-1C *hxt13Δ::loxP hxt15Δ::loxP hxt16Δ::loxP hxt14Δ::loxP hxt12Δ::loxP hxt9Δ::loxP hxt11Δ::loxP hxt10Δ::loxP hxt8Δ::loxP hxt514Δ::loxP hxt2Δ::loxP hxt367Δ::loxP gal2 Δ stl1Δ::loxP agt1Δ::loxP ydl247wΔ::loxP yjr160cΔ::loxP*
SC9721	MATa *his 3-D200 URA 3-52 leu2D1 lys 2D202 trp 1D63*
EBY.VW4000 +pRH195m +pRH274+pGH1	EBYVW4000 pRH195 pRH274pGH1
Tr69957::GFP:: EBY.VW4000	EBYVW4000 pRH195 *hxtB* pRH274
T. reesei	
QM6A	Δ*tmus53*Δ*Pyr4*
Δ*69957*	Δ*tmus53*Δ*Pyr4*Δ*69957*
**Plasmids**	
pRH195	pBluescript II SK+, *TRP1, CEN6, ARSH4+ PHXT7-XKS1-THXT7*
pRH274	pBluescript II SK+, *URA3, CEN6, ARSH4* + P*PGK*1-*XYL1*-T*PGK*1; P*ADH*1- *XYL2*-T*ADH*1; P*HXT*7-*XKS1*-T*HXT*7
pGH1	pRS425 *PGK1p-gh1-1-CYC1t*
