# Supplementary material for: Characterization of a novel sugar transporter involved in sugarcane bagasse degradation in Trichoderma reesei
Source: Biotechnol Biofuels. 2018 Apr 2;11:84. doi: 10.1186/s13068-018-1084-1 (PMC5879799; doi:10.1186/s13068-018-1084-1)
Supplement: Supplementary file 2 — Additional file 2. Oligonucleotides used for pyrG amplification, promoter region and terminator region of 69957. [file 13068_2018_1084_MOESM2_ESM.pdf]

Additional file 2. Oligonucleotides used for *pyrG* amplification, promoter region and terminator region of *69957*.

Oligonucleotides	Sequences
**Tr69957_pRS426_5fw**	5’GTAACGCCAGGGTTTTCCCAGTCACGACGGTGCTCAAGACATCCTAGGTATG-3’
**Tr69957_pyrG_5rv**	5’-CAGTGCCTCCTCTCAGACAGAATAGCAGTAGCGAAGTAGAGGCTGC-3’
**Tr69957_pyrG_3fw**	5´-GAGCATTGTTTGAGGCGAATTCAACCCACTTGACACCGTTGCCGG-3´
**Tr69957_pRS426_3rv**	5'-GCGGTTAACAATTTCTCTCTGGAAACAGCGCATCTGGGTGGTCGAGTAACG-3´
**pyrG_rv**	5’-GCAGCCTCTACTTCGCTACTGCTATTCTGTCTGAGAGGAGGCACTG-3’
**pyrG_fw**	5’-GAGCATTGTTTGAGGCGAATTCAACCCACTTGACACCGTTGCCGG-3’
