# Supplementary material for: Characterization of a novel sugar transporter involved in sugarcane bagasse degradation in Trichoderma reesei
Source: Biotechnol Biofuels. 2018 Apr 2;11:84. doi: 10.1186/s13068-018-1084-1 (PMC5879799; doi:10.1186/s13068-018-1084-1)
Supplement: Supplementary file 3 — Additional file 3. Southern blot analyse to confirm deletion of Tr69957 in T. reesei. (A) Strategy used to confirm deletion of Tr69957 by Southern blot. The figure shows the promoter region (in green) and terminator (in purple) of the gene, with the substitution of the ORF by the pyrG selection marker. A specific DNA probe was created to bind the portion of the gene (in blue), confirming its presence in the analyzed genetic content. (B) Southern blot showing the parental fungus (QM6aΔtmus53Δpyr4) used as control and four deleted candidates that did not present detection of the gene portion equivalent to 3391 bp as in the parental and presented the region corresponding to 6698bp, confirming the deletion. [file 13068_2018_1084_MOESM3_ESM.pdf]

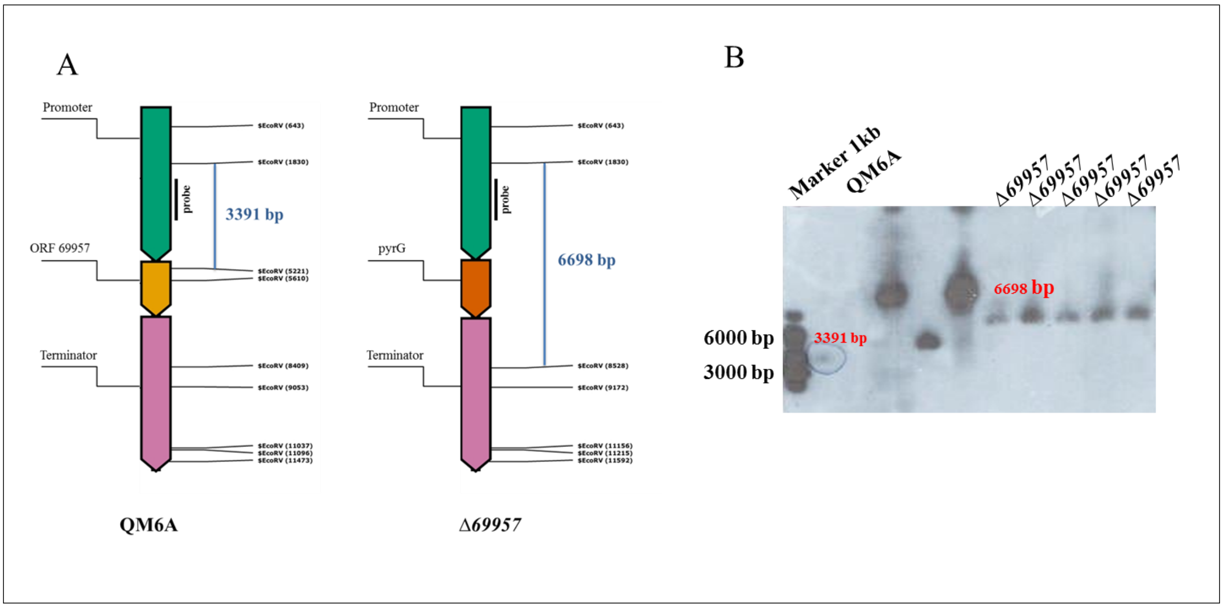

Additional file 3. Southern blot analyse to confirm deletion of *Tr69957* in *T. reesei*. (A) Strategy used to confirm deletion of *Tr69957* by Southern blot. The figure shows the promoter region (in green) and terminator (in purple) of the gene, with the substitution of the ORF by the *pyrG* selection marker. A specific DNA probe was created in order to bind the portion of the gene (in blue), confirming its presence in the analyzed genetic content. (B) Southern blot showing the parental fungus (QM6aΔ*tmus53*Δ*pyr4*) used as control and four deleted candidates that did not present detection of the gene portion equivalent to 3391 bp as in the parental and presented the region corresponding to 6698bp, confirming the deletion.
