# Supplementary material for: Characterization of a novel sugar transporter involved in sugarcane bagasse degradation in Trichoderma reesei
Source: Biotechnol Biofuels. 2018 Apr 2;11:84. doi: 10.1186/s13068-018-1084-1 (PMC5879799; doi:10.1186/s13068-018-1084-1)
Supplement: Supplementary file 4 — Additional file 4. Oligonucleotides used for evaluation of gene expression. [file 13068_2018_1084_MOESM4_ESM.pdf]

**Additional file 4.** Oligonucleotides used for evaluation of gene expression

| Oligonucleotides | Sequences |
|---|---|
| ***cel7a* F** | 5’ ACAAGAATGCATCGTCTCCG 3’ |
| ***cel7a* R** | 5’TGTTCCACCCGTTGTAGTTG 3’ |
| ***cel6a* F** | 5’ CCGAGCTTGGTAGTTACTCTG 3’ |
| ***cel6a* R** | 5’ GGTAGCCTTCTTGACTGAGT 3’ |
| **xyn1 F** | 5’GGC CAA ATT ATC GTC AAC TGT C 3’ |
| **xyn1 R** | 5’TCT GTC TTT TGG GCT TGG AG 3’ |
| **cel3a F** | 5’ CTG TAC ATC ACC TAC CCA TC3’ |
| **cel3a R** | 5’ TAG CTG AGA TCT CGT CGT C3’ |
| **cel1a F** | 5’ TTT GCC TGG TCG CTC ATG3’ |
| **cel1a R** | 5’ AAT CAG CTC GTC AAA CAG CG3’ |
| **69957 F** | 5' CTC TTA CAG TTG GCC CTA TCA C 3’ |
| **69957 R** | 5' GGT CGG GTT CAT AAA GTA CGG 3' |
| **xyn2 F** | 5’ TGT CAA CGA GCC TTC CAT C 3’ |
| **xyn2 R** | 5’ TCT GCA CAG TAA CAG TTC CG 3’ |
