# Supplementary material for: Characterization of a novel sugar transporter involved in sugarcane bagasse degradation in Trichoderma reesei
Source: Biotechnol Biofuels. 2018 Apr 2;11:84. doi: 10.1186/s13068-018-1084-1 (PMC5879799; doi:10.1186/s13068-018-1084-1)
Supplement: Supplementary file 5 — Additional file 5. Oligonucleotides used for complementation in S. cerevisiae. [file 13068_2018_1084_MOESM5_ESM.pdf]

Additional file 5. Oligonucleotides used for complementation in *S. cerevisiae*.

Oligonucleotides	Sequences
**pRH195_AN69957 START**	5´ GTTTTTTTAA TTTTAATCAA AATGGCGGAG GTCAACATC 3´
**ORF69957_SS_ GFP**	5' AGTTCTTCTCCTTTACTCATTCCCCGTGTTCCAGAA GAGG3’
**pRH195 GFP_CS_F- yeast**	5´ GAATTAATAAAAGTGTTCGCTTAACGCCAAGCTTGCATGC 3´
**spacerGFP 5´R_pRH195- yeast**	5’GGAACACGGG GA ATGAGTAA AGGAGAAGAA CTTTTCACTG G 3’
***69957* - 5F**	5’ GGGGGGATCCCTTTCGGAGGAACAAGTA 3’
***69957* - 3R**	5’GGGG ACTAGTTGTTTTCTCTCTGCCAGTTG 3’
